# Preoperative Cancer Antigen 125 Level as Predictor for Complete Cytoreduction in Ovarian Cancer: A Prospective Cohort Study and Systematic Review

**DOI:** 10.3390/cancers14235734

**Published:** 2022-11-22

**Authors:** Puck E. Brons, Gatske M. Nieuwenhuyzen-de Boer, Christian Ramakers, Sten Willemsen, Malika Kengsakul, Heleen J. van Beekhuizen

**Affiliations:** 1 Department of Gynecologic Oncology, Erasmus MC Cancer Institute, University Medical Center Rotterdam, 3000 CA Rotterdam, The Netherlands; 2 Department of Obstetrics and Gynecology, Albert Schweitzer Hospital, 3318 AT Dordrecht, The Netherlands; 3 Department of Clinical Chemistry, Erasmus MC, 3015 GD Rotterdam, The Netherlands; 4 Department of Epidemiology, Erasmus MC, 3015 GD Rotterdam, The Netherlands; 5 Department of Biostatistics, Erasmus MC, 3015 GD Rotterdam, The Netherlands; 6 Department of Obstetrics and Gynecology, Panyananthaphikkhu Chonprathan Medical Center, Srinakharinwirot University, Nonthaburi 11120, Thailand

**Keywords:** CA-125, predictor, epithelial ovarian cancer, cytoreductive surgery, surgical outcome

## Abstract

**Simple Summary:**

Standard treatment of advanced stage epithelial ovarian cancer consists of cytoreductive surgery (CRS) and chemotherapy. Prolonged progression free and overall survival is correlated with the amount of residual tumor after CRS. However, cytoreductive surgery is an extensive procedure with a considerable risk of postoperative complications. Therefore, it would be valuable to have parameters that can predict the surgical outcome. In this study, we evaluated the value of the blood tumor marker CA-125 to predict complete CRS to no residual tumor. The results of our study suggest that CA-125 levels ≤35 kU/L significantly predict the surgical outcome in patients who underwent interval cytoreductive surgery, but this parameter cannot be used as an independent predictor. This contrasts with the outcome of our systematic review with mainly retrospective data, which found CA-125 as an independent predictive variable.

**Abstract:**

Background: The tumor marker ‘cancer antigen 125’ (CA-125) plays a role in the management of women with advanced stage ovarian cancer. This study aims to describe the predictive value of pre-treatment CA-125 level and the reduction after neoadjuvant chemotherapy (NACT) on surgical outcome. Methods: A systematic review and a prospective clinical study were performed. Multiple databases were searched from database inception to April 2022. The clinical study is part of a randomized controlled trial named “PlaComOv-study”. A regression analysis was performed to demonstrate correlations between preoperative CA-125 levels, CA-125 reduction after NACT, and surgical outcome. Results: Fourteen relevant articles were analyzed of which eleven reported that lower preoperative CA-125 levels were associated with a higher probability of complete cytoreduction. In the clinical study, 326 patients with FIGO stage IIIB-IV ovarian cancer who underwent CRS were enrolled from 2018 to 2020. Patients who underwent interval CRS with preoperative CA-125 levels ≤35 kU/L had higher odds of achieving complete CRS than patients with CA-125 level >35 kU/L (85% vs. 67%, OR 2.79, 95%CI 1.44–5.41, *p* = 0.002). In multivariable analysis with presence of ascites and peritoneal carcinomatosis, normalized preoperative CA-125 did not appear as a significant predictor for complete CRS. Conclusions: In literature, preoperative CA-125 levels ≤35 kU/L were associated with a significant higher percentage of complete CRS in univariable analysis. According to our cohort study, preoperative CA-125 level ≤35 kU/L cannot independently predict surgical outcome either for primary or interval CRS.

## 1. Introduction

The tumor marker ‘cancer antigen 125’ (CA-125) plays a role in the management of women with advanced stage ovarian cancer. For those patients, the cornerstone treatment is the combination of cytoreductive surgery (CRS) and chemotherapy. How much value can we give to the CA-125 level to predict surgical outcome, and can we use CA-125 to decide to start neoadjuvant chemotherapy (NACT) or to perform a primary CRS? More than 300,000 women worldwide were diagnosed with epithelial ovarian cancer (EOC) in 2020. Unfortunately, 75% of them were initially diagnosed with advanced stage [1,2].

CA-125 is a tumor marker that is elevated in 80–90% of patients with EOC [3,4]. Patients with EOC will commonly undergo blood CA-125 analysis preceding treatment. Previous studies have shown that a lower level of preoperative CA-125 was associated with a higher probability of achieving optimal CRS [5,6,7]. During the past several years, the definition of ‘optimal cytoreduction’ after CRS has been changed from a maximum residual tumor diameter of 3 cm to one less than 1 cm [8]. Currently, we aim to achieve a complete CRS without any macroscopic residual disease when performing either primary or interval CRS.

The predictive value of CA-125 level for complete CRS is still inconclusive. Furthermore, we do not know whether normalization of CA-125 is a predictor of complete CRS or whether a particular drop can also be used as a predictor of surgical outcome.

To help fill this knowledge gap, we performed a systematic review and a prospective cohort study. The aim of this study was to demonstrate whether we can use CA-125 as a predictor of complete CRS for patients with advanced stage EOC. The secondary aim was to determine the predictive value of the CA-125 reduction rate after neoadjuvant chemotherapy on the surgical outcome for interval CRS.

## 2. Materials and Methods

### 2.1. Systematic Review

#### 2.1.1. Search Strategy

This review is reported in accordance with the Preferred reporting items for systematic reviews and meta-analyses (Prisma) Checklist [9], and the Prisma-S extension to the PRISMA Statement for Reporting Literature Searches in Systematic Reviews [10]. Embase.com, Medline Ovid, Web of Science Core Collection, Cochrane Central Register of Trials, and Google Scholar from which the most relevant 50 references were included, were searched from database inception to April 2022. The search strategy contained terms for (1) CA-125 (2) ovarian cancer and (3) CRS. The search strategy is presented in Supporting Information Supplementary Table S1.

#### 2.1.2. Eligibility Criteria

The key inclusion criteria were patients with International Federation of Gynecology and Obstetrics (FIGO) stage IIIB-IV ovarian cancer, English-language studies, complete surgical outcome, the correlation of preoperative CA-125 level or the changes of CA-125 level after NACT with surgical outcome. Exclusion criteria were patients with FIGO stage I-IIA ovarian cancer, recurrence of disease, and an optimal or suboptimal CRS or survival as primary outcome.

#### 2.1.3. Data Extraction

The retrieved electronic citations were de-duplicated, and the titles and abstracts were screened on relevance for the review independently by two researchers (PB and GN) using EndNote X9. Subsequently, the full texts of eligible citations were screened for relevance to the review. Any disagreements between both researchers were resolved through discussion. The reference lists of retrieved articles were searched for possibly missed relevant studies. Extracted data included FIGO staging, type of CRS (primary or interval), surgical outcome, level of CA-125 and reduction of CA-125 after NACT (when applicable), and optimal cut-off value of CA-125.

#### 2.1.4. Quality Assessment

Quality of the included studies was assessed with the Quality In Prognosis Studies (QUIPS) tool [11]. Risk of study bias in studies of prognostic factors was appraised in five important domains: study participation, prognostic factor measurement, outcome measurement, study confounding, statistical analysis, and reporting. Each domain was rated and then classified as low, moderate, or high risk of bias. Low risk means least risk of bias: results are generally considered valid, there is clear description of population (>100), setting, inclusion criteria, interventions, comparison groups, and statistics are clearly defined.

Moderate risk means susceptible risk to some bias: studies may not meet all the criteria for the low risk of bias rating, but do not show flaws that could cause major bias. The study population is 50–100 people. The study may also lack information, making it difficult to assess limitations and potential problems. High risk means significant deficiencies that may invalidate the results: deficiencies in design, analysis or reporting, small sample size, large amounts of missing information, and discrepancies in reporting.

### 2.2. Prospective Study

The second part of the study consisted of a prospective cohort study within the framework of a multicenter randomized controlled trial named “PlaComOv-study” [12]. The aim of PlaComOv-study was to evaluate the effectiveness of the PlasmaJet surgical device in the treatment of advanced stage ovarian cancer. Participants were randomized to the additional use of the PlasmaJet surgical device during surgery or only the conventional surgical devices. The study was conducted in four gynecological oncology centers and nine centers specialized in ovarian cancer surgery in the Netherlands. The standard chemotherapy regimen given after primary CRS consisted of six cycles of intravenously carboplatin (area under the curve of 6 mg per milliliter per minute) and paclitaxel (175 mg per square meter of body-surface area) with an interval of three weeks for each cycle. The regimen for interval CRS consisted of three cycles of NACT given prior to surgery and three cycles given after surgery in all cases [13].

#### 2.2.1. Inclusion and Exclusion Criteria

Patients with suspected advanced stage EOC, fallopian tube or peritoneal carcinoma FIGO stage IIIB-IV who were fit enough to undergo CRS and chemotherapy were eligible for inclusion. The surgical procedure was either primary CRS or interval CRS. Subjects were included in the study if advanced stage EOC (FIGO IIIB-IV) was pathologically reported. Patients were excluded with recurrent disease, a non-epithelial, borderline ovarian tumor, ovarian metastasis of another primary tumor, as well as patients who did not have surgery after randomization because of their comorbidity. Informed consent was obtained from all subjects involved in the PlaComOv-study.

#### 2.2.2. Data Collection

Clinical characteristics and operative reports of the participants of the PlaComOv-study were stored in an electronic database management platform called ‘Open Clinica’. The preoperative levels of blood CA-125 were related to type of surgery, either primary or interval CRS. Normal CA-125 level was defined as <35 kU/L, as this cut-off value is used in general practice [14]. The reduction of CA-125 level after NACT was calculated and presented as a percentage. Blood CA-125 level was determined in 13 different laboratories using five different automated immunochemistry platforms. While all assays use the same general analytical principle (automated sandwich assays with monoclonal capture), the antigen-binding sites of the antibodies as well as the signal technology for quantifying tumor markers concentrations may be disparate. The detailed methods of each platform used to analyze CA-125 are presented in Supporting Information Supplementary Table S2.

The surgical outcome was classified in four categories: complete CRS, optimal CRS, suboptimal CRS, and unresectable disease. Complete CRS was defined as no macroscopic residual disease after surgery. Optimal CRS was defined as residual tumor lesions of ≤1 cm. Suboptimal CRS was defined as residual tumor lesions >1 cm. Unresectable disease was defined as surgery with the intention to perform CRS, but which the surgeon stopped when after exploration resection proved not possible.

In addition, preoperative CA-125 levels were categorized as ≤35 kU/L vs. >35 kU/L. The reduction of CA-125 level after NACT among patients who underwent interval CRS was categorized as ≤95% change vs. >95% change.

### 2.3. Statistical Analyses

Continuous variables were analyzed using the one-way ANOVA or the Kruskal–Wallis test. Categorical variables were compared using the chi-squared test. Univariable analysis with binary logistic regression was conducted to compare variables between categories of CA-125 level as well as between categories of surgical outcome. Multivariable analysis with binary logistic regression was conducted to identify variables independently predicting complete CRS. All variables in univariable analysis were included in the multivariable model. ROC analysis with cross-validation was performed to evaluate the combined value of significant predictors of complete CRS. Cross-validation was applied, and it was considered that ROC curves were created with a selection of variables. Differences with a two-tailed *p*-value of *p* < 0.05 were considered statistically significant. All statistical analyses were performed in IBM SPSS Statistics Version 25 (SPSS Inc., Chicago, IL, USA).

## 3. Results

### 3.1. Systematic Review

#### 3.1.1. General Characteristics of the Studies

**Figure 1:**
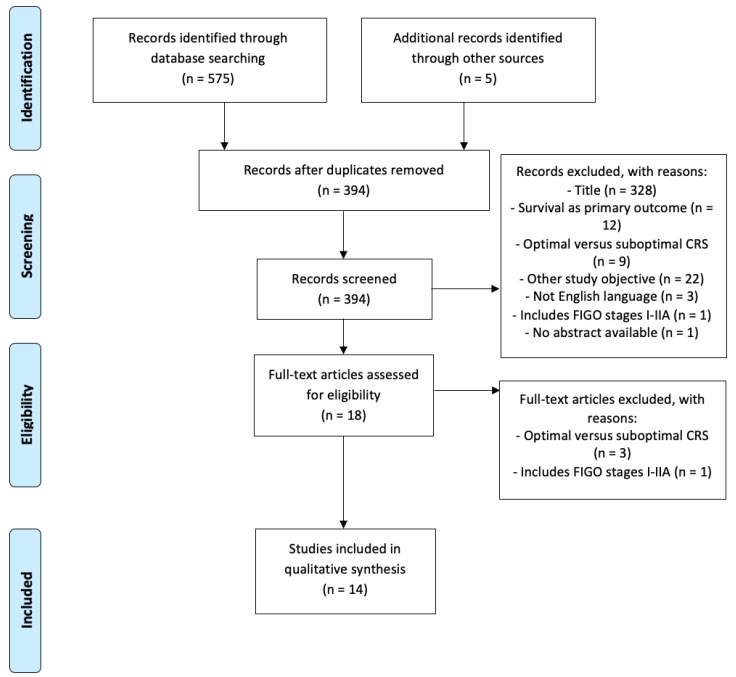
Flowchart of study selection.

The searches revealed 580 potentially relevant articles. After excluding duplicates and screening on relevance, 14 articles met the inclusion criteria (Figure 1). Records were excluded because of the following reasons: conference paper, editorials, letter and short communications, not ovarian malignancy, not English language, recurrence of ovarian cancer, survival as primary outcome, optimal versus suboptimal CRS, and inclusion of FIGO stage I-IIA. The QUIPS quality assessment outcomes of the included studies are reported in Supporting Information Supplementary Table S3. Generally, the risk of bias was low for most studies [8,15,16,17,18,19,20,21,22,23,24]. The risk of bias was moderate for only three studies [25,26,27].

The characteristics of the included studies are summarized in Table 1. Two studies were prospective cohort studies [15,25]; the other twelve studies were retrospective cohort studies [8,16,17,18,19,20,21,22,23,24,26,27]. Four out of 14 studies reported the results of patients who underwent primary CRS [15,16,17,25], and nine studies reported the results of patients who underwent interval CRS [8,18,19,20,21,22,23,24,26]. One study reported patients undergoing either primary or interval CRS [27]. Generally, patients were diagnosed with FIGO stage IIIA-IV EOC [10,24,26–36), and the majority of patients in all studies were diagnosed with a serous tumor.

#### 3.1.2. Primary Cytoreductive Surgery and CA-125 Level

The study of Risum et al. compared preoperative CA-125 levels between patients with complete CRS vs. non-complete CRS. The authors demonstrated that patients who underwent complete CRS had significantly lower preoperative CA-125 levels in (*p* = 0.03) [25]. Eltabbakh et al. reported a significant effect of lower preoperative CA-125 levels on complete CRS in univariable analysis, but not in multivariable analysis [17]. Merlo et al. reported a higher rate of complete CRS in patients with lower preoperative CA-125 levels [27]. Karlsen et al. and Jung et al. reported CA-125 level as part of a prediction model of factors predicting surgical outcome of CRS. While Karlsen et al. excluded preoperative CA-125 levels from their prediction model after multivariable analysis. Jung et al. reported that high preoperative levels of CA-125 were correlated with non-complete CRS (*p* = 0.001). The authors found that the use of preoperative CA-125 level, computed tomography (CT) factor scores, and surgical skill resulted in a C-statistic of 0.73 (95%CI 0.67–0.79) [15,16].

#### 3.1.3. Interval Cytoreductive Surgery and CA-125 Level

Five studies reported a significant relation between lower preoperative CA-125 levels and a complete CRS rate after interval CRS [21,22,24,26,27]. Matsuhashi et al. reported a significant relation between lower preoperative CA-125 levels and complete/optimal CRS (*p* = 0.003) [19]. Gupta et al. demonstrated higher rates of complete CRS in patients with preoperative levels of CA-125 <100 kU/L (*p* = 0.00) [18], whereas Zeng et al. reported the cut off level of CA-125 level ≤200 kU/L (*p* = 0.012) [8]. In two studies, preoperative CA-125 was not a significant predictor of surgical outcome in multivariable analysis [20,23]. Additionally, patients in the studies received NACT before surgery, varying from 1–14 cycles. Only one study reported 3 cycles before interval CRS for all patients [21].

Eight studies demonstrated the effect of preoperative levels of CA-125 change after NACT on surgical outcome [8,18,19,20,21,22,23,27]. After adjusting for potential confounders, Gupta et al. found a significant correlation between >95% decrease rate of preoperative CA-125 and complete CRS [18]. Pelissier et al. also reported a significant relation between a level of CA-125 after three cycles of NACT and complete CRS in multivariable analysis [19]. Furthermore, Merlo et al. reported an association between >96.4% reduction of CA-125 after NACT and complete CRS [26]. Nevertheless, other studies did not find the decrease in CA-125 level after NACT to be an independent predictor for complete CRS [8,19,21,22,23]. Ten studies reported an optimal cut-off value of CA-125 level, either prior to primary CRS or interval CRS [8,17,18,19,20,21,22,23,26,27]. The cut-off values of preoperative CA-125 ranged from ≤20 kU/L [21] to ≤500 kU/L [17,27].

### 3.2. Prospective Study

#### 3.2.1. Patient Characteristics and Surgical Outcomes

**Figure 2:**
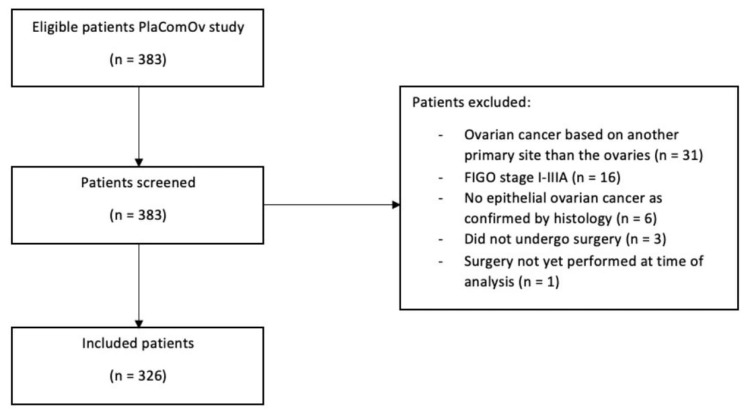
Flowchart of patient selection.

Of the 383 patients randomized for the PlaComOv-study between 2018 and 2020, 326 patients were included in our study (Figure 2). Patients’ baseline characteristics were analyzed according to the intention-to-treat protocol and are presented in Table 2. Complete CRS was achieved in 71.8% (*n* = 234). The majority of patients had been diagnosed with FIGO stage IIIB/IIIC (69.3%) and serous histology (96.0%). Non-seroius tumors were mucinous, endometrioid, clear cell, mixed epithelial, or carcinosarcoma. The PlasmaJet surgical device was used in 119 patients (50.9%) with complete CRS, in 12 patients (24.5%) with optimal CRS and in eight patients (50.0%) with suboptimal CRS (*p* = 0.002). In case of unresectable disease, cytoreductive surgery was omitted. The extent of surgery required to achieve the outcome was also documented. These results can be found in the original article of the PlaComOv-study [28].

#### 3.2.2. Primary Cytoreductive Surgery and CA-125 Level

A total of 45 patients (13.8%) underwent primary CRS. The median preoperative level of CA-125 was 297.4 kU/L in patients with complete CRS, 171.0 kU/L in patients with optimal CRS, and 185.9 kU/L in patients with suboptimal CRS (*p* = 0.868). All four patients with CA-125 level ≤35 kU/L at time of primary cytoreductive surgery had a complete cytoreduction. However, the level of preoperative CA-125 did not show an association with surgical outcome in primary CRS (Supporting Information Supplementary Table S4).

#### 3.2.3. Interval Cytoreductive Surgery and CA-125 Level

A total of 281 patients (86.2%) underwent interval CRS. Among these patients, the median preoperative CA-125 was 67.0 kU/L in those with a complete CRS, 77.5 kU/L in those with an optimal CRS, and 252.5 kU/L in those with a suboptimal CRS (*p* = 0.026). The median percentage of CA-125 reduction after NACT was 91.1% in the complete CRS group, 89.9% in the optimal CRS group, 78.0% in the suboptimal CRS group, and 88.6% in the unresectable disease group (*p* = 0.388).

#### 3.2.4. Reduction of CA-125 Level after NACT

Supporting information in Supplementary Table S5 demonstrates factors associated with the normalization of CA-125 level after 3 cycles of NACT. In 85 patients (30.8%) who received NACT, the levels of CA-125 decreased to less than 35 kU/L. Having ascites and/or peritoneal carcinomatosis significantly decreased the chance of having a CA-125 reduction to normal level, (12.9% vs. 36.6%; OR 0.25, 95%CI 0.13–0.50, *p* < 0.001)) and (23.5% vs. 48.7%; OR 0.31, 95%CI 0.18–0.55, *p* < 0.001)) respectively. When using a 95% decrease in CA-125 after NACT [18], no significant factors were found (OR 1.28, 95%CI 0.71–2.31, *p* = 0.42). In 30% of the patients, the level of CA-125 had been reduced >95% between diagnosis and preoperative interval CRS (Supporting Information Supplementary Table S6).

#### 3.2.5. Multivariable Analysis

Table 3 shows the univariable and multivariable analysis of factors predicting a complete interval CRS. Preoperative CA-125 levels ≤35 kU/L before interval CRS significantly predicted complete CRS (OR 2.79, 95%CI 1.44–5.41, *p* = 0.002). Furthermore, perioperative absence of ascites and peritoneal carcinomatosis both significantly predicted complete CRS in univariable analysis (*p* < 0.001). In multivariable analysis, patients with normalized level of CA-125 had higher odds of complete CRS than patients with elevated level of CA-125 (OR 1.74, 95%CI 0.74–4.09, *p* = 0.207). The absence of ascites (*p* < 0.001) and peritoneal carcinomatosis (*p* < 0.001) independently predicted complete CRS. Other significant independent predictors were FIGO stage IIIB/IIIC (*p* = 0.002), a seroius histology (*p* = 0.013) and the additional use of the PlasmaJet during surgery (*p* = 0.026).

#### 3.2.6. ROC Analysis

ROC curves were created with preoperative CA-125 level <35 kU/L, FIGO staging, histology, perioperative presence of ascites and peritoneal carcinomatosis, and with the use of PlasmaJet. The area under the curve (AUC) was 0.876 for primary CRS. After applying cross-validation, the mean AUC was 0.801 (Supplementary Figure S1). For interval CRS, the overall AUC was 0.837. After applying cross-validation, the mean AUC was 0.798 (Supplementary Figure S2).

## 4. Discussion

The results of our systematic review indicated a significant relation between lower preoperative level of CA-125 and complete CRS in patients who underwent an interval CRS [8,18,19,21,22,24,26,27]. Studies of patients who underwent a primary CRS demonstrated an inconsistent correlation between the CA-215 level and surgical outcome [15,16,17,25,27]. It must be noted, however, that definitions of the normal value of CA-125 and optimal cut-off values of CA-125 reduction varied among studies in the literature, which implies that the effect of CA-125 reduction rate after NACT on the surgical outcome could not be directly compared between the existing studies.

In our prospective cohort study of 326 patients, normalized values of CA-125 in patients receiving interval CRS showed a significant relation with complete CRS in univariable analysis. Complete CRS was achieved in 84.7% (*n* = 72) of patients CA-125 levels ≤ 35 kU/L, whereas this was only 66.5% (*n* = 127) of patients with CA-125 levels > 35 kU/L. However, only the absence of ascites and peritoneal carcinomatosis during surgery, FIGO stage IIIB/IIIC and serious histology were the significant independent predictors of complete interval CRS as presented in multivariable analysis. Furthermore, use of the PlasmaJet surgical device was an independent predictor of complete CRS, which is in line with the results of the PlaComOv study [12,28].

The studies included in our systematic review did not report the effect of ascites or peritoneal carcinomatosis [5,6,7,8,9,10,11,12,14,15,16,17,18,19,20,21,22,23,25,27,29,30,31,32,33,34,35,36], and lacked a multivariable analysis [19,24,25,26,27]. Fortunately, the risk of bias was generally low among most studies. Our finding was in contrast to that of Gupta et al., in that we did not find a significant relation between >95% decrease in CA-125 level after NACT and complete CRS [18]. Surprisingly, in patients who underwent primary CRS, preoperative CA-125 level did not appear as a significant predictor for complete cytoreduction, although 73.3% of the patients underwent complete cytoreduction and all patients with normal preoperative CA-125 levels had a complete CRS. This is probably because the population of patients with primary CRS was only 14% of our cohort. However, we expect interval CRS to be performed more frequently in the future, as studies have shown that overall survival is similar to that of primary CRS and there tends to be a decreased risk of postoperative complications [13]. Therefore, we still decided to include this patient group in our research.

The results of our clinical study and some studies in the systematic review are remarkably inconsistent, likely due to selection bias or missing data due to the retrospective nature of most of the included studies. It is quite possible that other studies that have determined the value of CA-125 on surgical outcome, but which have not shown significant outcomes, have not been published. Furthermore, in our clinical study, we have focused on a CA-125 cut-off value of 35 kU/L. This value is generally accepted as cut-off point [14]. We additionally applied cut-off values suggested in our systematic review to our study population, but these were found to be non-significant. Moreover, we found no supporting evidence in the literature that the proposed cut-off values are valuable.

We performed both a carefully constructed systematic review through multiple databases and a clinical investigation. Conducting a meta-analysis was not possible since we could not obtain raw data from each study. The clinical data of our cohort have been prospectively collected for the PlaComOv-study. All variables in the univariable analysis were selected for the multivariable logistic regression to identify all independent predictors of complete CRS. The use of the PlasmaJet surgical device, which has been reported to increase the rate of complete CRS [28], was evenly distributed among all groups.

Nevertheless, some limitations need to be addressed. First, CA-125 levels of patients who were not eligible for interval CRS were lacking because of the progressive disease during chemotherapy or because of health deterioration. Second, ROC analysis implied that the combination of significant independent predictors and CA-125 is an acceptable prediction model. However, since CA-125 ≤35 kU/L was not a significant predictor in multivariable analysis, we do not know the added value of this variable in the prediction model. Third, the participating hospitals used different methods to measure the level of CA-125 in each. The reliability of CA-125 results depends on precision and trueness, where trueness is of particular importance for the comparability of CA-125 results between laboratories and hospitals. In general, immuno assays, including tumor marker assays, are poorly standardized resulting in a marginal trueness. This is caused in part by differences in antibody-antigen binding characteristics which can differ significantly per used method. During our analyses, we compared the different methods of CA-125 determination. This made no difference to the described results. Therefore, we decided to include all patients in the study to be able to analyze a larger group. It is striking that the articles included in the systematic review do not provide a description of the way in which the CA-125 has been determined in their method. Therefore, we advise researchers considering follow-up research to use standardized tumor marker assays and to describe this clearly in their methods section.

## 5. Conclusions

In conclusion, the value of CA-125 as a preoperative predicting factor for surgical outcome of primary CRS in advanced stage EOC is inconclusive. Still, the normalization of CA-125 after NACT (≤35 kU/L) was significantly associated with a higher percentage of complete CRS in patients who underwent interval CRS, in line with the literature. Nevertheless, CA-125 level for surgical outcome prediction should be used with caution. Preoperative CA-125 level should not be used as an isolated predictive parameter.

## Figures and Tables

**Table 1:** Summary of study characteristics.

Authors	Journal, Publication Year	Sample Size	FIGO Staging	Primary Outcome Measures	Type of CRS	CA-125 Reduction after NACT	Surgical Outcome	Number of NACT Cycles	Results for Preoperative CA-125	Results for CA-125 Reduction after NACT	Optimal CA-125 Cut-Off Value	Additional Information
Eltabbakh et al. [14]	Gynecol Oncol, 2004	72	15.3% Stage IIIA 5.6% Stage IIIB 61.1% Stage IIIC 18.1% Stage IV	Relation between preoperative CA-125 and surgical outcome	Primary CRS	N/A	Complete vs. non-complete CRS	N/A	In univariable analysis, preoperative levels of CA-125 predict complete CRS (*p* < 0.001). In multivariable analysis, preoperative CA-125 was not significantly associated with complete CRS.	N/A	≤500 kU/L preoperative	No or small amount of ascites significantly correlated with complete CRS (*p* < 0.001) in univariable analysis. In multivariable analysis, the only independent predictor of complete CRS was p53 expression (*p* < 0.001).
Risum et al. [22]	Int J Gynecol Cancer, 2009	75	86.7% Stage III 13.3% Stage IV	The predictive value of preoperative CA-125 on incomplete CRS	Primary CRS	N/A	Complete vs. non-complete CRS	N/A	Preoperative CA-125 levels are significantly lower in patients undergoing complete CRS (*p* = 0.03).	N/A	N/A	Intestinal carcinosis was found in 92% of incomplete CRS and in only 13% of complete CRS.
Rodriguez et al. [19]	Gynecol Oncol, 2012	103	40.5% Stage IIIC 59.5% Stage IV	Relation between change of CA-125 in patients undergoing NACT and surgical outcome	Interval CRS	Overall percentage change, >80% change from presentation to operation and prior to each NACT cycle	Complete vs. optimal CRS	Median: 3 cycles, Range: 1–8 cycles	Lower preoperative CA-125 levels, especially ≤100 kU/L, significantly correlate with complete CRS (*p* = 0.04).	No significant relation between decrease in CA-125 during NACT and complete CRS.	≤100 kU/L preoperative	
Furukawa et al. [18]	J Gynecol Oncol, 2013	75	6.7% Stage IIIA 93.3% Stage IIIC	Relation between CA-125 after NACT and surgical outcome Relation between changes of CA-125 levels during NACT and surgical outcome	Interval CRS	Rates of changes prior to each NACT cycle	Complete vs. non-complete CRS	3 cycles: 100%	Preoperative CA-125 levels are significantly lower in patients undergoing complete CRS (*p* < 0.001) in both univariable and multivariable analysis.	In univariable analysis, (pre-NACT CA-125—pre-2nd NACT CA-125)/pre-NACT CA-125 (*p* = 0.01) and (pre-NACT CA-125—pre-3rd NACT CA-125)/pre-NACT CA-125 (*p* = 0.008) significantly predicted complete CRS. In multivariable analysis, there was no significant relation between decrease in CA-125 during NACT and complete CRS.	≤20 kU/L preoperative	
Jung et al. [12]	Gynecol Oncol, 2013	358	12.1% Stage IIC 71.5% Stage III 15.1% Stage IV 0.3% undocumented	Developing a model to predict non-complete CRS with CA-125, CT scan, age and surgical skill index	Primary CRS	N/A	Complete vs. non-complete CRS	N/A	Higher preoperative CA-125 levels significantly predict non-complete CRS (*p* = 0.001).	N/A	N/A	CA-125 (*p* = 0.001), two CT factor scores and surgical skill index were included in the model.
Pelissier et al. [17]	Gynecol Oncol, 2014	148	72.3% Stage IIIC 23.6% Stage IV	Relation between kinetic CA-125 levels during NACT and surgical outcome	Interval CRS	Percentage decrease in CA-125	Complete vs. non-complete CRS	Median: 6 cycles, Range: 1–9 cycles	In univariable analysis, preoperative CA-125 (*p* = 0.001) significantly predicts complete CRS, but not according to multivariable analysis.	In univariable analysis, level of CA-125 after 3 cycles of NACT (*p* = 0.00001), cycle to nadir (*p* = 0.001) and percentage decrease (*p* = 0.01) significantly predict complete CRS. Multivariable analysis shows that only CA-125 after 3 cycles of NACT independently predicts complete CRS (*p* = 0.04).	<75 kU/L after 3 cycles of NACT	
Karlsen et al. [11]	Tumor Biol, 2016	150	78.7% Stage IIIC 21.3% Stage IV	Relation between preoperative CA-125, HE4, age, presence of ascites and performance status and the surgical outcome	Primary CRS	N/A	Complete vs. non-complete CRS	N/A	In univariable analysis, preoperative CA-125 are significantly lower in patients undergoing complete CRS (*p* = 0.001).	N/A	N/A	CA-125 was excluded from the prediction model (no significant contribution to the model (*p* = 0.166)). Included in the model were: age, HE4 and performance status.
Morimoto et al. [23]	Jpn J Clin Oncol, 2016	139	59.7% Stage IIIC 40.3% Stage IV (Primary ovarian, fallopian tube and peritoneal)	Relation between CA-125 after NACT, presence of ascites and response rate and the surgical outcome	Interval CRS	N/A	Complete vs. non-complete CRS	Median: 4 cycles Range: 3–6 cycles	Preoperative CA-125 levels are significantly lower in patients undergoing complete CRS (*p* < 0.001).	N/A	≤25.8 kU/L preoperative	Presence of preoperative ascites leads to a significant lower complete CRS rate (*p* < 0.0001).
Zeng et al. [8]	J Cancer, 2016	118	84.7% Stage III 15.3% Stage IV (Primary ovarian, fallopian tube and peritoneal)	Relation between preoperative CA-125 and surgical outcome Relation between changes in CA-125 during NACT and surgical outcome	Interval CRS	Percentage reduction after the first NACT cycle and >30% reduction, overall percentage reduction, >80% reduction after all NACT cycles	Complete vs. non-complete CRS	1–3 cycles: 97.5% ≥4 cycles: 2.5%	In univariable analysis, preoperative value of CA-125 ≤200 kU/L (*p* = 0.000) predicts complete CRS. This was also significant in multivariable analysis (*p* = 0.012).	In univariable analysis, >80% reduction of CA-125 after NACT (*p* = 0.000) predicts complete CRS. This was not significant in multivariable analysis (*p* = 0.059).	≤200 kU/L preoperative	
Matsuhashi et al. [16]	J Nippon Med Sch, 2017	107	55.1% Stage III 44.9% Stage IV	Relation between CA-125 after NACT and surgical outcome	Interval CRS	Number of NACT cycles needed for CA-125 levels to halve or reduce to <35 U/mL	Complete/optimal vs. suboptimal CRS	6 cycles: >70%, ≤5 cycles: <30%	Lower preoperative CA-125 levels (*p* = 0.003), especially <35 kU/L (*p* = 0.0029), significantly correlate with complete/optimal CRS.	No significant difference in frequency of NACT cycles for CA-125 levels to halve or to drop below 35 U/mL between complete/optimal vs. suboptimal CRS (*p* > 0.05).	<35 kU/L preoperative	
Ghisoni et al. [20]	J Ovarian Res, 2018	93	9.7% Stage IIIA 15% Stage IIIB 62.4% Stage IIIC 12.9% Stage IV	Developing a predictive score of cytoreductive outcome	Interval CRS	<96% reduction in CA-125	Complete vs. non-complete CRS	N/A *	In univariable analysis, preoperative CA-125 >33 kU/L significantly predicted non-complete CRS (*p* = 0.002). This was not significant in multivariable analysis.	In univariable analysis, <96% reduction in CA-125 after NACT significantly predicted non-complete CRS (*p* = 0.034). This was not significant in multivariable analysis.	<33 kU/L preoperative >96% reduction	Age >60 years (*p* = 0.007), CA-125 at diagnosis ≥550 kU/L (*p* = 0.014) and peritoneal cancer index assessed at laparoscopy of >16 (*p* < 0.001) were included in the prediction model.
Gupta et al. [15]	South Asian J Cancer, 2020	406	71.5% Stage III 28.5% Stage IV	Relation between preoperative CA-125 and surgical outcome Relation between percent fall of CA-125 after NACT and surgical outcome	Interval CRS	>95% vs. <95% decrease in CA-125	Complete vs. optimal and vs. suboptimal CRS	<3 cycles: >60%, >3 cycles: <40%	Rate of complete CRS is significantly higher in preoperative levels of CA-125 <100 kU/L (*p* = 0.00).	Rate of complete CRS is significantly higher in >95% fall of CA-125 after NACT (*p* = 0.00).	<100 kU/L preoperative >95% decrease in CA-125 after NACT	Mucinous carcinomas were excluded.
Nakamura et al. [21]	World J Surg Oncol, 2020	63	68.3% Stage IIIC 31.7% Stage IV	This study aimed to use CA-125 and CT scanning to generate a model of predicting complete cytoreduction	Interval CRS	N/A	Complete vs. non-complete CRS	Median: 6 cycles Range: 1–14 cycles	Pre-operative levels of CA-125 were significantly lower in patients with complete CRS (*p* = 0.015).	N/A	N/A	Extra-ovarian implants (*p* = 0.009) and omental tumors at CT after NACT (*p* = 0.038) are significantly associated with complete CRS.
Merlo et al [24]	Radiol Oncol, 2021	253	Primary CRS: 89.5% Stage IIIC 10.5% Stage IV Interval CRS: 66.6% Stage IIIC 33.4% Stage IV	Relation between pre-operative CA-125 and surgical outcome	Primary CRS and Interval CRS	Percentage reduction	Complete vs. optimal and vs. suboptimal CRS	N/A *	Primary CRS: Lower preoperative levels of CA-125 are associated with complete/optimal CRS. Interval CRS: Lower preoperative levels of CA-125 are associated with complete/optimal CRS (*p* = 0.020).	The probability of complete/optimal CRS is higher in patients with a CA-125 reduction >96.4%.	<500 kU/L preoperative	

CRS = cytoreductive surgery, CT = computed tomography, FIGO = international federation of gynecology and obstetrics, N/A = not applicable, NACT = neoadjuvant chemotherapy. * = not mentioned in the article.

**Table 2:** Patients’ characteristics (*N* = 326).

Characteristics	Complete CRS (*n* = 234, 71.8%)	Optimal CRS (*n* = 49, 15.0%)	Suboptimal CRS (*n* = 16, 4.9%)	Unresectable Disease (*n* = 27, 8.3%)	*p*-Value
Median AGE (IQR), years	65 (15)	67 (9)	66 (18)	72 (12)	0.038
Median BMI (IQR), kg/m^2^	24.7 (6.2)	24.5 (5.6)	24.5 (3.7)	22.5 (7.9)	0.424
WHO performance, *n* (%)					NS
0	130 (55.6)	26 (53.1)	7 (43.8)	9 (33.3)	
1	75 (32.1)	17 (34.7)	4 (25.0)	13 (48.2)	
≥2	14 (5.9)	3 (6.1)	4 (25.0)	4 (14.8)	
TYPE OF SURGERY, N (%)					NS
PRIMARY CRS	33 (14.1)	8 (16.3)	4 (25.0)	0 (0.0)	
INTERVAL CRS	201 (85.9)	41 (83.7)	12 (75.0)	27 (100.0)	
FIGO stage, *n* (%)					0.082
IIIB/IIIC	169 (72.2)	28 (57.1)	13 (81.3)	16 (59.3)	
IV	65 (27.8)	21 (42.9)	3 (18.7)	11 (40.7)	
Histology, *n* (%)					NS
Serous	226 (96.6)	46 (93.9)	14 (87.5)	27 (100.0)	
Non-serous	8 (3.4)	3 (6.1)	2 (12.5)	0 (0.0)	
Randomization: PlasmaJet, *n* (%)	119 (50.9)	12 (24.5)	8 (50.0)	N/A	0.002
Peritoneal carcinomatosis on CT, *n* (%)	159 (67.9)	34 (69.4)	12 (75.0)	19 (70.4)	0.940
Presence of ascites, *n* (%)	60 (25.6)	19 (38.8)	11 (68.8)	23 (85.2)	<0.001
Median ascites volume (IQR), mL	100 (350)	250 (400)	300 (450)	250 (400)	0.578
Presence of peritoneal carcinomatosis, *n* (%)	65 (27.8)	34 (69.4)	12 (75.0)	24 (88.9)	<0.001
Primary CRS					
Median preoperative CA-125 (IQR), kU/L	297.4 (602.3)	171.0 (388.2)	185.9 (1828.6)	N/A	0.868
Interval CRS					
Median preoperative CA-125 (IQR), kU/L	67.0 (178.0)	77.5 (166.8)	252.5 (532.5)	81.0 (209.0)	0.026
Median percentage of CA-125 reduction after NACT (IQR), %	91.1 (21.8)	89.9 (13.8)	78.0 (44.0)	88.6 (40.2)	0.388

BMI = body mass index, CA-125 = cancer antigen 125, CRS = cytoreductive surgery, FIGO = international federation of gynecology and obstetrics, IQR = interquartile range, N/A = not applicable, NS = non-significant, WHO = world health organization.

**Table 3:** Univariable and multivariable analysis of factors predicting complete interval CRS.

Factor	Univariable		Multivariable	
	OR [95%CI]	*p*-Value	OR [95%CI]	*p*-Value
Median AGE (IQR), years	0.99 [0.97–1.01]	0.417	1.00 [0.96–1.03]	0.768
Median BMI (IQR), kg/m^2^	1.03 [0.98–1.09]	0.226	1.07 [0.99–1.16]	0.092
WHO performance status, ***n*** (%)				
0 (ref)				
1	0.71 [0.42–1.22]	0.214	1.10 [0.53–2.30]	0.800
≥2	0.41 [0.17–0.98]	0.044	0.54 [0.16–1.80]	0.318
FIGO stage, *n* (%)				
IIIB/IIIC (ref)				
IV	0.63 [0.38–1.04]	0.072	0.29 [0.14–0.63]	0.002
Histology, ***n*** (%)				
Serous (ref)				
Non-serous	0.62 [0.20–1.93]	0.407	0.12 [0.02–0.64]	0.013
Randomization: PlasmaJet, ***n*** (%)	1.47 [0.90–2.40]	0.121	2.26 [1.10–4.61]	0.026
Presence of peritoneal carcinomatosis on CT, ***n*** (%)	0.88 [0.52–1.49]	0.636	1.05 [0.49–2.27]	0.893
Presence of ascites, ***n*** (%)	0.25 [0.15–0.42]	<0.001	0.26 [0.13–0.55]	<0.001
Presence of peritoneal carcinomatosis, ***n*** (%)	0.11 [0.06–0.19]	<0.001	0.10 [0.05–0.23]	<0.001
Median preoperative CA-125, kU/L	1.00 [1.00–1.00]	0.990	1.00 [1.00–1.00]	0.628
Preoperative CA-125 ≤ 35 kU/L	2.79 [1.44–5.41]	0.002	1.74 [0.74–4.09]	0.207

CA-125 = Cancer Antigen 125, CI = confidence interval, CRS = cytoreductive surgery, IQR = interquartile range, OR = odds ratio, REF = reference.

## Data Availability

Data are available on a reasonable request to corresponding author.

## Supplementary Materials

The following supporting information can be downloaded at: https://www.mdpi.com/article/10.3390/cancers14235734/s1, Table S1: The searching strings, Table S2: Detailed methods of each platform used to analyze CA-125, Table S3: Univariable analysis of factors predicting normal levels of preoperative CA-125 in primary CRS, Table S4: Univariable analysis of factors predicting normalized preoperative CA-125 level before interval CRS, Table S5: Univariable analysis of factors predicting >95% reduction in CA-125 after NACT. Table S6: Univariable analysis of factors predicting >95% reduction in CA-125 after NAC. Figure S1: cross-validated ROC-curve for primary CRS, Figure S2: cross-validated ROC-curve for interval CRS.

## Abbreviations

CA-125	Cancer Antigen 125
CRS	Cytoreductive Surgery
CT	Computed Tomography
EOC	Epithelial Ovarian Cancer
FIGO	International Federation of Gynecology and Obstetrics
NACT	Neoadjuvant Chemotherapy
WHO	World Health Organization
