# Supplementary material for: Preoperative Cancer Antigen 125 Level as Predictor for Complete Cytoreduction in Ovarian Cancer: A Prospective Cohort Study and Systematic Review
Source: Cancers (Basel). 2022 Nov 22;14(23):5734. doi: 10.3390/cancers14235734 (PMC9740757; doi:10.3390/cancers14235734)
Supplement: Supplementary file 1 [file cancers-14-05734-s001.zip › cancers-2002178-supplementary.pdf]

## Supporting information CA-125 manuscript

## Table S1. The searching strings

| Database | |
|---|---|
| **Embase.com** | ('ovary carcinoma'/exp OR ('ovary tumor'/de AND ('carcinoma'/de OR 'adenocarcinoma'/de)) OR (((epithelial* OR mucinous* OR serous* OR endometrioid*) NEAR/6 (ovar*) NEAR/6 (carcinoma* OR adenocarcinoma*))):ab,ti,kw) AND ('cytoreductive surgery'/de OR (cytoreduc* OR debulking*):ab,ti,kw) AND ('CA 125 antigen'/de OR 'cancer antigen 125'/de OR 'carcinoembryonic antigen'/de OR (ca-125 OR ca125 OR cancer-antigen-125 OR cancer-antigen125 OR carcinoembryonic-antigen* OR CEA OR Mucin-16*):ab,ti,kw) AND (((advanced OR 2b OR 2c OR 3 OR 3a OR 3b OR 3c OR 4 OR iib OR iic OR iii OR iiia OR iiib OR iiic OR iv OR 2-b OR 2-c OR 3-a OR 3-b OR 3-c OR ii-b OR ii-c OR iii-a OR iii-b OR iii-c) NEAR/3 (stag*))):ab,ti,kw NOT [conference abstract]/lim |
| **Medline Ovid** | (Carcinoma, Ovarian Epithelial/ OR (((epithelial* OR mucinous* OR serous* OR endometrioid*) ADJ6 (ovar*) ADJ6 (carcinoma* OR adenocarcinoma*))).ab,ti,kf.) AND (Cytoreduction Surgical Procedures/ OR (cytoreduc* OR debulking*).ab,ti,kf.) AND (CA-125 Antigen/ OR (ca-125 OR ca125 OR cancer-antigen-125 OR cancer-antigen125 OR carcinoembryonic-antigen* OR CEA OR Mucin-16*).ab,ti,kf.) AND (((advanced OR 2b OR 2c OR 3 OR 3a OR 3b OR 3c OR 4 OR iib OR iic OR iii OR iiia OR iiib OR iiic OR iv OR 2-b OR 2-c OR 3-a OR 3-b OR 3-c OR ii-b OR ii-c OR iii-a OR iii-b OR iii-c) ADJ3 (stag*))).ab,ti,kf. NOT (letter* OR news OR comment* OR editorial* OR congres* OR abstract* OR book* OR chapter* OR dissertation abstract*).pt. |
| **Web of science** | TS=(((((epithelial* OR mucinous* OR serous* OR endometrioid*) NEAR/5 (ovar*) NEAR/5 (carcinoma* OR adenocarcinoma*)))) AND ((cytoreduc* OR debulking*)) AND ((ca-125 OR ca125 OR cancer-antigen-125 OR cancer-antigen125 OR carcinoembryonic-antigen* OR CEA OR Mucin-16*)) AND (((advanced OR 2b OR 2c OR 3 OR 3a OR 3b OR 3c OR 4 OR iib OR iic OR iii OR iiia OR iiib OR iiic OR iv OR 2-b OR 2-c OR 3- |

| | |
|---|---|
| | a OR 3-b OR 3-c OR ii-b OR ii-c OR iii-a OR iii-b OR iii-c) NEAR/2 (stag*)))) |
| **Cochrane Central** | ((((epithelial* OR mucinous* OR serous* OR endometrioid*) NEAR/6 (ovar*) NEAR/6 (carcinoma* OR adenocarcinoma*))):ab,ti,kw) AND ((cytoreduc* OR debulking*):ab,ti,kw) AND ((ca NEXT 125 OR ca125 OR cancer NEXT antigen NEXT 125 OR cancer NEXT antigen125 OR carcinoembryonic NEXT antigen* OR CEA OR Mucin NEXT 16*):ab,ti,kw) AND (((advanced OR 2b OR 2c OR 3 OR 3a OR 3b OR 3c OR 4 OR iib OR iic OR iii OR iiia OR iiib OR iiic OR iv OR 2 NEXT b OR 2 NEXT c OR 3 NEXT a OR 3 NEXT b OR 3 NEXT c OR ii NEXT b OR ii NEXT c OR iii NEXT a OR iii NEXT b OR iii NEXT c) NEAR/3 (stag*))):ab,ti,kw |
| **Google Scholar** | "epithelial ovarian carcinoma\|adenocarcinoma" cytoreductive\|debulking "ca 125"\|ca125\|"carcinoembryonic antigen"\|CEA "advanced\|2b\|2c\|3a\|3b\|3c\|4 stage\|staging" |

## Table S2. Detailed methods of each platform used to analyze CA-125

| | Roche | Abbott | Beckman Coulter | Siemens | Thermo Fischer |
|---|---|---|---|---|---|
| **Platform** | E601, E801 | Architect i2000 | DxI 800 | Centaur | Kryptor Compact |
| **Method** | ECLIA | MEIA | CLEIA | CLEIA | TRACE |
| **Signal technology** | Ruthenium | AP | AP | Acrid-Fluor | Cryp-Acc |

ECLIA = Electro-Chemiluminescent Immuno Assay; MEIA = Microparticle Enzyme Immuno Assay; CLEIA = Chemiluminiscent Enzyme Immuno Assay; AP = Alkaline Phosphatase; Acrid-Fluor = Acrdinium-Fluorescein; TRACE = Time Resolved Amplified Cryptate Emission; Cryp-Acc = Cryptate-Acc

## Table S3. Outcome of quality assessment

| | Study participation | Prognostic factor measurement | Outcome measurement | Study confounding | Statistical analysis and reporting | Total |
|---|---|---|---|---|---|---|
| *Eltabbakh et al.*[25] | Green | Yellow | Green | Green | Green | Green |
| *Risum et al.*[33] | Yellow | Yellow | Green | Yellow | Yellow | Yellow |
| *Rodriguez et al.*[30] | Green | Green | Green | Green | Yellow | Green |
| *Furukawa et al.*[29] | Yellow | Green | Green | Green | Green | Green |
| *Jung et al.*[24] | Green | Yellow | Green | Yellow | Green | Green |
| *Pelissier et al.*[28] | Green | Green | Green | Green | Green | Green |
| *Karlsen et al.*[23] | Green | Green | Green | Green | Green | Green |
| *Morimoto et al.*[34] | Green | Yellow | Yellow | Yellow | Green | Yellow |
| *Zeng et al.*[6] | Green | Green | Green | Green | Green | Green |
| *Matsuhashi et al.*[27] | Green | Green | Yellow | Yellow | Green | Green |
| *Ghisoni et al.*[31] | Green | Yellow | Yellow | Green | Green | Green |
| *Gupta et al.*[26] | Green | Green | Green | Green | Yellow | Green |
| *Nakamura et al.*[32] | Green | Yellow | Green | Yellow | Green | Green |
| *Merlo et al.*[35] | Green | Yellow | Green | Yellow | Yellow | Yellow |

Green = low risk of bias; Yellow = moderate risk of bias

## Table S4. Univariable analysis of factors predicting normal levels of preoperative CA-125 before primary CRS

| | Preoperative CA-125 ≤35 kU/L (n=4, 9.1%) | Preoperative CA-125 >35 kU/L (n=40, 90.9%) | Unadjusted OR [95%CI] | *P*-value |
|---|---|---|---|---|
| Median AGE (IQR), years | 66 (22) | 59 (17) | 1.07 [0.96-1.20] | 0.208 |
| Median BMI (IQR), kg/m$^2$ | 22.2 (7.5) | 24.8 (6.7) | 0.89 [0.69-1.15] | 0.373 |
| WHO performance, n (%) | | | | |
| 0 (ref) | 4 (100.0) | 25 (62.5) | | |
| 1 | 0 (0.0) | 11 (27.5) | N/A* | N/A* |
| ≥2 | 0 (0.0) | 3 (7.5) | N/A* | N/A* |
| FIGO stage, n (%) | | | | |
| IIIB/IIIC (ref) | 4 (100.0) | 37 (92.5) | | |
| IV | 0 (0.0) | 3 (7.5) | N/A* | N/A* |
| Histology, n (%) | | | | |
| Serous (ref) | 3 (75.0) | 39 (97.5) | | |
| Non-serous | 1 (25.0) | 1 (2.5) | 13.00 [0.64-263.82] | 0.095 |
| Presence of peritoneal carcinomatosis on CT, n (%) | 2 (50.0) | 22 (55.0) | 0.82 [0.11-6.40] | 0.848 |
| Presence of ascites, n (%) | 1 (25.0) | 30 (75.0) | 0.11 [0.01-1.19] | 0.070 |
| Median ascites (IQR), ml | 20 (-) | 400 (700) | 0.94 [0.76-1.17] | 0.570 |
| Presence of peritoneal carcinomatosis, n (%) | 1 (25.0) | 18 (45.0) | 0.41 [0.04-4.26] | 0.453 |
| Surgical outcome, n (%) | | | | |
| Complete CRS | 4 (100.0) | 28 (70.0) | N/A* | N/A* |
| Non-complete CRS (ref) | 0 (0.0) | 12 (30.0) | | |

CA-125 = Cancer Antigen 125, CI = confidence interval, CRS = cytoreductive surgery, IQR = interquartile range, N/A = not applicable, OR= odds ratio, REF = reference

* = odds-ratio or *P*-value could not be calculated

## Table S5. Univariable analysis of factors predicting normalized preoperative CA-125 level before interval CRS

| | Preoperative CA-125 ≤35 kU/L (n=85, 30.8%) | Preoperative CA-125 >35 kU/L (n=191, 69.2%) | Unadjusted OR [95%CI] | *P*-value |
|---|---|---|---|---|
| Median AGE (IQR), years | 68 (14) | 67 (12) | 1.00 [0.98-1.03] | 0.884 |
| Median BMI (IQR), kg/m$^2$ | 24.6 (5.8) | 24.5 (6.0) | 1.00 [0.95-1.06] | 0.905 |
| WHO performance, n (%) | | | | |
| 0 (ref) | 48 (60.0) | 93 (52.0) | | |
| 1 | 29 (36.3) | 67 (37.4) | 0.84 [0.48-1.466] | 0.536 |
| ≥2 | 3 (3.8) | 19 (10.6) | 0.31 [0.09-1.09] | 0.067 |
| FIGO stage, n (%) | | | | |
| IIIB/IIIC (ref) | 56 (65.9) | 124 (64.9) | | |
| IV | 29 (34.1) | 67 (35.1) | 0.96 [0.56-1.64] | 0.877 |
| Histology, n (%) | | | | |
| Serous (ref) | 84 (98.8) | 183 (95.8) | | |
| Non-serous | 1 (1.2) | 8 (4.2) | 0.27 [0.03-2.21] | 0.224 |
| Presence of ascites/peritoneal carcinomatosis on CT, n (%) | 57 (67.1) | 140 (73.3) | 0.74 [0.43-1.29] | 0.291 |
| Presence of ascites, n (%) | 11 (12.9) | 70 (36.6) | 0.25 [0.13-0.50] | <0.001 |
| Median ascites (IQR), ml | 100 (185) | 200 (300) | 1.00 [0.99-1.00] | 0.228 |
| Presence of peritoneal carcinomatosis, n (%) | 20 (23.5) | 93 (48.7) | 0.31 [0.18-0.55] | <0.001 |
| Surgical outcome, n (%) | | | | |
| Complete CRS | 72 (84.7) | 127 (66.5) | 2.79 [1.44-5.41] | 0.002 |
| Non-complete CRS (ref) | 13 (15.3) | 64 (33.5) | | |

CA-125 = Cancer Antigen 125, CI = confidence interval, CRS = cytoreductive surgery, IQR = interquartile range, OR= odds ratio, REF = reference

## Table S6. Univariable analysis of factors predicting >95% reduction in CA-125 after NAC

| | >95% decrease in preoperative CA-125 (n=81, 29.6%) | ≤95% decrease in preoperative CA-125 (n=193, 70.4%) | Unadjusted OR [95%CI] | *P*-value |
|---|---|---|---|---|
| Median AGE (IQR), years | 66 (15) | 68 (12) | 0.98 [0.96-1.01] | 0.251 |
| Median BMI (IQR), kg/$m^2$ | 24.5 (5.3) | 24.6 (6.3) | 0.97 [0.91-1.02] | 0.228 |
| WHO performance, n (%) | | | | |
| 0 (ref) | 50 (61.7) | 89 (46.1) | | |
| 1 | 23 (28.4) | 73 (37.8) | 0.56 [0.31-1.00] | 0.052 |
| ≥2 | 4 (4.9) | 18 (9.3) | 0.40 [0.13-1.23] | 0.110 |
| FIGO stage, n (%) | | | | |
| IIIB/IIIC (ref) | 50 (61.7) | 128 (66.3) | | |
| IV | 31 (38.3) | 65 (33.7) | 1.22 [0.71-2.09] | 0.467 |
| Histology, n (%) | | | | |
| Serous (ref) | 79 (97.5) | 186 (96.4) | | |
| Non-serous | 2 (2.5) | 7 (3.6) | 0.67 [0.14-3.31] | 0.626 |
| Presence of peritoneal carcinomatosis on CT, n (%) | 57 (70.4) | 138 (71.5) | 0.95 [0.54-1.67] | 0.850 |
| Presence of ascites, n (%) | 15 (18.5) | 66 (34.2) | 0.44 [0.23-0.83] | 0.011 |
| Median ascites (IQR), ml | 200 (200) | 200 (300) | 1.00 [1.00-1.001] | 0.459 |
| Presence of peritoneal carcinomatosis, n (%) | 30 (37.0) | 83 (43.0) | 0.77 [0.45-1.32] | 0.347 |
| Surgical outcome, n (%) | | | | |
| Complete CRS | 61 (75.3) | 136 (70.5) | 1.28 [0.71-2.31] | 0.416 |
| Non-complete CRS (ref) | 20 (24.7) | 57 (29.5) | | |

CA-125 = Cancer Antigen 125, CI = confidence interval, CRS = cytoreductive surgery, IQR = interquartile range, OR= odds ratio, REF = reference

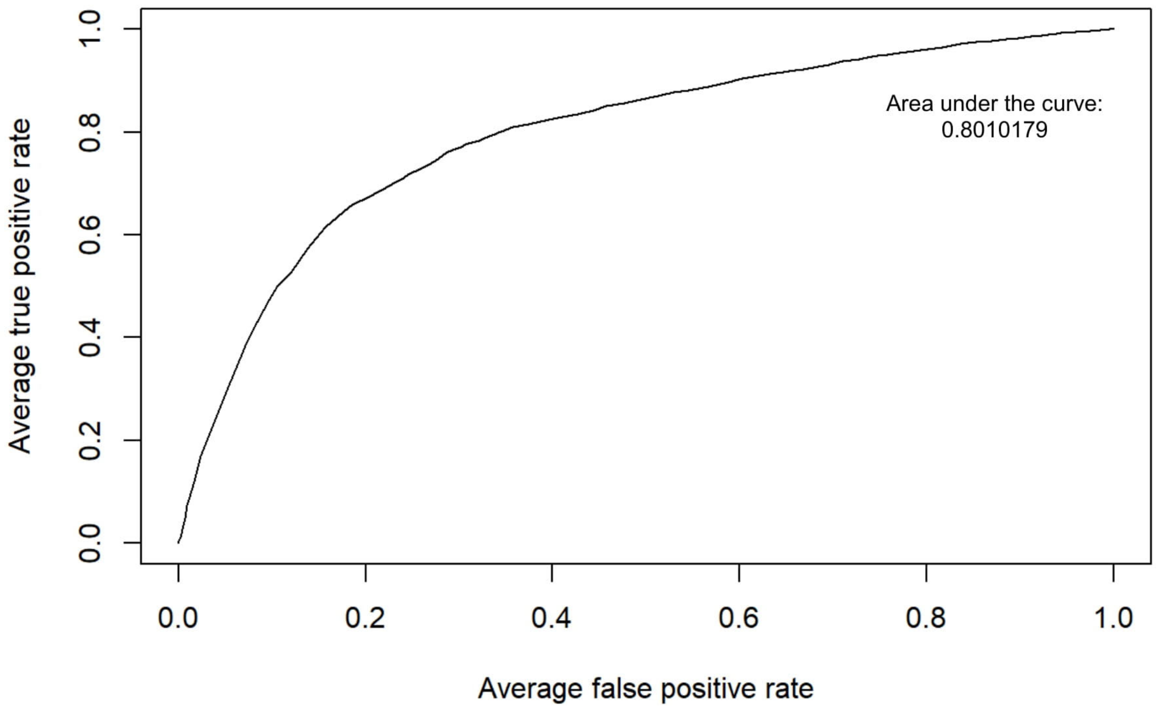


Figure S1. cross-validated ROC-curve for primary CRS.

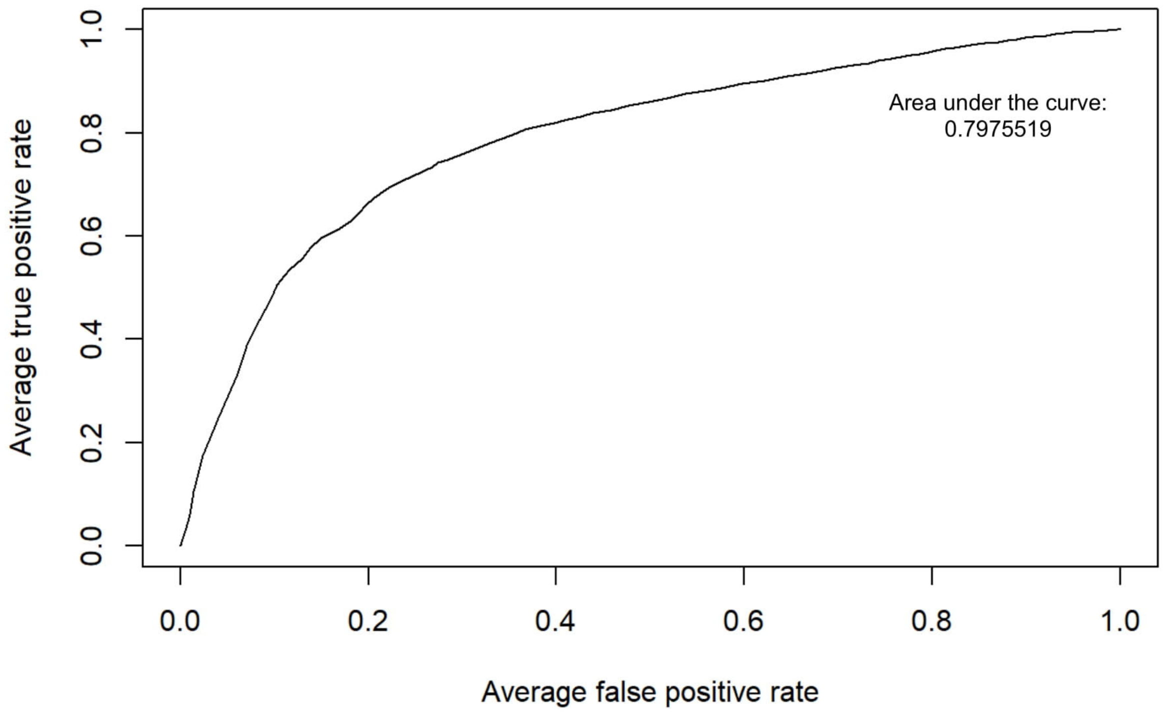


Figure S2: cross-validated ROC-curve for interval CRS.
